# Loss of ASD-Related Molecule Caspr2 Affects Colonic Motility in Mice

**DOI:** 10.1101/2023.04.17.537221

**Published:** 2023-04-19

**Authors:** Beatriz G. Robinson, Beau A. Oster, Keiramarie Robertson, Julia A. Kaltschmidt

**Affiliations:** 1 Wu Tsai Neurosciences Institute, Stanford University, Stanford, CA 94305, USA; 2 Neurosciences IDP Graduate Program, Stanford University School of Medicine, Stanford, CA 94305, USA; 3 Nevada ENDURE Program, University of Nevada, Reno, Reno, NV 89557, USA; 4 Department of Neurosurgery, Stanford University School of Medicine, Stanford, CA 94305, USA

**Keywords:** Autism spectrum disorder, gastrointestinal dysmotility, Caspr2, enteric nervous system, sensory neurons

## Abstract

Gastrointestinal (GI) symptoms are highly prevalent among individuals with autism spectrum disorder (ASD), but the molecular link between ASD and GI dysfunction remains poorly understood. The enteric nervous system (ENS) is critical for normal GI motility and has been shown to be altered in mouse models of ASD and other neurological disorders. Contactin-associated protein-like 2 (Caspr2) is an ASD-related synaptic cell-adhesion molecule necessary for regulating sensory function in the central and peripheral nervous system. In this study, we examine the role of Caspr2 in GI motility by characterizing Caspr2’s expression in the ENS and assessing ENS organization and GI function in *Caspr2* mutant mice. We find that Caspr2 is predominantly expressed in enteric sensory neurons in both the small intestine and colon. We further assess colonic motility in *Caspr2* mutants using an *ex-vivo* motility monitor and show altered colonic contractions and faster expulsion of artificial pellets. The organization of neurons within the myenteric plexus remains unchanged. Our results suggest a role for enteric sensory neurons in ASD-related GI dysmotility, a finding relevant to consider in the treatment of ASD-related GI symptoms.

## Lay Summary

Autism spectrum disorder (ASD) patients experience sensory abnormalities and chronic gastrointestinal (GI) problems. We ask if the ASD-related synaptic cell-adhesion molecule Caspr2*,* associated with ASD-related hypersensitivity in the central and peripheral nervous system, is present and/or plays a role in GI function in mice. Results show Caspr2 is present in enteric sensory neurons; lack of Caspr2 alters GI motility, suggesting enteric sensory dysfunction may contribute to ASD-related GI symptoms.

## Introduction:

Autism spectrum disorder (ASD) is a neurodevelopmental disorder currently affecting approximately 1 in 44 children in the USA (Maenner et al., 2021). Patients with ASD often report gastrointestinal (GI) issues, which can lead to irritability and social withdrawal, ultimately affecting their quality of life (Chaidez et al., 2014; Restrepo et al., 2020; Valicenti-McDermott et al., 2006). GI issues, including constipation, diarrhea, and abdominal pain (Chaidez et al., 2014; Holingue et al., 2018), have been correlated with sensory over-responsivity in the central and peripheral nervous system in children with ASD (Mazurek et al., 2013). Whether intrinsic sensory functions of the GI tract are affected in ASD, and thus might potentially trigger GI dysfunction has not yet been explored.

The ENS is a quasi-autonomous neuronal network that populates the length of the GI tract and can regulate GI function and motility independent of the central nervous system (CNS) (Furness et al., 1994). Neurons and glia cluster into ganglia that reside within the myenteric and submucosal plexus within the gut wall (Sasselli et al., 2012). GI motility initiates when intrinsic enteric sensory neurons, known as intrinsic primary afferent neurons (IPANs), are activated by chemical or mechanical stimuli. IPANs signal to enteric interneurons that stimulate excitatory or inhibitory motor neurons, resulting in the contraction and relaxation of GI muscles (Fung & Vanden Berghe, 2020; Furness et al., 1994; Rao & Gershon, 2016) and propulsive motility referred to as a migrating motor complex (MMC) (Corsetti et al., 2019). Based on the sensory dysfunctions observed in patients with ASD, coupled with the high prevalence of constipation and diarrhea, we hypothesize that dysfunction of enteric sensory neurons might contribute to ASD-related GI issues.

Several synapse-associated genes have been studied in relation to GI function. A mutation in zebrafish, *shank3,* which encodes a synapse-associated protein critical for synaptic transmission, results in a reduction of serotonin-expressing enteroendocrine cells and reduced GI transit (James et al., 2019). In mice, a global deletion of Nlgn3, an ASD-related synaptic cell adhesion molecule, results in increased colonic diameter and faster colonic MMCs (CMMCs) (Leembruggen et al., 2020). Here we study Contactin-associated protein-like 2 (Caspr2), an ASD-related presynaptic cell-adhesion molecule that aids in forming trans-synaptic complexes in the central and peripheral nervous system (Gordon et al., 2016; Peñagarikano et al., 2011; Peñagarikano & Geschwind, 2012; Poliak et al., 1999). *Caspr2*^*−/−*^ mice have altered neural circuitry in the somatosensory cortex and exhibit hypersensitivity to mechanical stimuli due to enhanced excitability of primary dorsal root afferents (Dawes et al., 2018; Gordon et al., 2016; Peñagarikano et al., 2011). Additionally, in the GI tract, *CASPR2* loss of function has been associated with inflammatory bowel disease in humans, and *Caspr2*^*−/−*^ mice are reported to have increased intestinal permeability (Buniello et al., 2019; Graf et al., 2019). These findings highlight the potential importance of Caspr2 in regulating overall GI function.

In this study, we assess Caspr2 expression in the adult mouse ENS and ask whether ENS organization and GI motility are altered in *Caspr2*^*−/−*^ mice. We find that Caspr2 is expressed in IPANs in both small intestine (SI) and colon, being nearly exclusive to IPANs in the colon. We further assess colonic motility in an *ex-vivo* motility monitor and show that lack of Caspr2 leads to altered CMMCs and faster expulsion of artificial pellets. The overall organization of the ENS appears undisturbed.

## Material and Methods:

### Animals

All procedures conformed to the National Institutes of Health Guidelines for the Care and Use of Laboratory Animals and were approved by the Stanford University Administrative Panel on Laboratory Animal Care. C57BL/6, B6.129(Cg)-Cntnap2^tm1Pele^/J (Strain #:017482, hereafter *Caspr2*^*−*^), and B6.129(Cg)-Cntnap2^tm2Pele^/J (Strain #:028635, hereafter *Caspr2*^*tlacZ*^) mice were purchased from The Jackson Laboratory (Bar Harbor, ME). Mice were maintained on a 12:12 LD cycle. Food and water were provided *ad libitum* and mice were group housed with a maximum of five adults per cage. Both male and female 8–12 week-old adult mice were used in this study.

### Histology

Mice were euthanized by CO_2_ followed by cervical dislocation. Segments of jejunum and colon were dissected from *Caspr2*^*−/−*^ mice and *Caspr2*^*WT*^ littermate controls, flushed with cold PBS, and cut longitudinally along the mesenteric border. Segments were opened flat, placed between sheets of filter paper, and immersed in 4% PFA at 4°C for 90 min. Tissue was rinsed three times in PBS for 10 min and immersed in a 30% sucrose solution overnight at 4°C. Tissue sections were rolled into a “swiss-roll” preparation as described (Williams et al., 2016), embedded in OCT (Tissue-Tek), and frozen until use. 14 μm slices were sectioned using a Leica Cryostat (Leica CM3050 S) and mounted on Superfrost glass slides. Slides were stained with hematoxylin and eosin (H&E). Brightfield images were taken by the Human Pathology/Histology Service Center at Stanford School of Medicine and analyzed for villus height, crypt depth, colonic fold thickness, and circular muscle thickness using Leica ImageScope software. Villus height was measured when full lacteal was visible and crypt depth was measured when both villus/crypt junctions were present in the jejunum. Colonic fold thickness was measured from cross sections of mid and distal colon. 10 measurements were taken per animal. To determine muscle thickness, we took 20 measurements at random points along the length of the jejunum and distal colon.

### Tissue Dissection and Processing

Dissection and tissue processing of the intestines was performed as previously described (Hamnett et al., 2022). Wholemount muscle-myenteric plexus preparations were made by peeling away the muscularis (longitudinal and circular muscle with myenteric plexus). The tissue was stored in PBS with 0.1% sodium-azide at 4°C for up to 3 weeks. Jejunum samples were taken from the middle 1/3 length of the SI. The final 1/3 of the colon was considered the distal colon.

### Immunohistochemistry

Segments of the jejunum (≥1 cm in length) and distal colon (≥0.5 cm in length) were used for immunohistochemistry studies. Staining was performed as previously described (Hamnett et al., 2022), with modifications. For cell body labeling with anti-Caspr2 antibody, PBT contained 0.01% Triton X-100; for all other labeling, PBT contained 0.1% Triton X-100. Primary antibodies used included rabbit anti-Caspr2 (1:1000; Alomone Labs, APZ-005), rabbit anti-ß-galactosidase (1:1000; gift from J. Sanes), human anti-HuC/D (ANNA1) (1:100,000; gift from V. Lennon), goat anti-Sox10 (1:2,000; R&D Systems, AF2864) and fluorophore-conjugated secondary antibodies (Jackson Labs and Molecular Probes).

### RNAscope *In Situ* Hybridization with Protein Co-detection

Tissue was dissected and prepared for fixation as outlined in *Tissue Dissection and Processing*. Flat segments of the jejunum and distal colon were fixed overnight in 4% PFA. Segments were rinsed with PBS, and wholemount muscle-myenteric plexus preparations were made by peeling away the muscularis. RNAscope *in situ* with protein co-detection was performed using Advanced Cell Diagnostics (ACD) RNAscope Multiplex Fluorescent Reagent Kit v2 (Cat# 323100) and ACD RNA-protein Co-detection ancillary kit (Cat# 323180) as described in (Guyer et al., 2023). The following RNAscope probes were used: Mm-Nmu-C1 (Cat# 446831) and Mm-Cntnap2-C1 (Cat# 449381).

### Neuron Quantification

Images were acquired on a Leica SP8 confocal microscope using 20x and 63x oil objectives. All images were adjusted for brightness and contrast using ImageJ/FIJI. For Caspr2 quantification, three 20x ROIs (1000 μm × 1000 μm) per mouse were randomly selected in both the jejunum and distal colon. HuC/D^+^ and Caspr2^+^ neurons were counted manually using the cell counter tool FIJI (Schindelin et al., 2012). For each region, neurons per ROI were averaged for each animal.

For quantification of Caspr2 co-expression with *Nmu* transcript, five images (138 μm × 138 μm) were taken at 63x magnification per region per mouse. Regions of interest (ROIs) were created around every HuC/D^+^ neuron for each image and manually scored as either positive or negative for Caspr2 or *Nmu* transcript. Neurons with ≥20 *Nmu* fluorescent transcript dots were considered positive. For each region, the average percentage of co-expression was calculated per mouse.

Quantification of ganglia was performed using COUNTEN (Kobayashi et al., 2021), with *σ* = 4.5. For each region, the average of three maximum projection images (1000 μm × 1000 μm) were analyzed per mouse

### Functional Behavior

#### Whole GI transit time

Whole GI transit assay was performed as previously described (Spear et al., 2018). In brief, mice were gavaged with a carmine red-methylcellulose mixture and observed until a red pellet was expelled.

#### Gastric Emptying and SI Transit

Gastric emptying and SI transit were determined as previously described (De Lisle, 2007; Spear et al., 2018). In brief, mice were fasted for 12 hours and water was removed 3 hours before the start of the assay. Mice were gavaged with a 2% methylcellulose mixture containing 2.5 mg/mL Rhodamine B Dextran (Invitrogen, D1841, MW: 70,000). 15 minutes after gavage, mice were euthanized with CO_2_ and the stomach and SI were removed. The SI was divided into 10 equal segments that were homogenized in saline. The fluorescence in the stomach and each SI segment was measured. The percentage of gastric emptying and the geometric center were determined as described (De Lisle, 2007).

#### Bead Expulsion Assay

Bead expulsion assay was performed as previously described (Spear et al., 2018). In brief, mice were lightly anesthetized by isoflurane and a 2 mm glass bead was inserted 2 cm into the colon through the anus using a gavage needle. Expulsion time was determined as the time from bead insertion to when the bead was fully expelled.

#### Fecal Water Content & Pellet Length

Fecal water content was assessed as previously described (Spear et al., 2018) with modifications to allow for measurement of pellet lengths. Mice were housed individually for 1 hour during which all fecal pellets were collected immediately after expulsion, photographed, and stored in a pre-weighed tube (1 tube/mouse). After 1 hour of collection, tubes were weighed again, incubated for 48 hours at 50°C, and weighed a final time to determine the percentage of water content. Pellet length was measured using FIJI.

#### *Ex-vivo* Colonic Motility Assay

*Ex-vivo* motility monitor assay was adapted from (Hennig et al., 1999; Spear et al., 2018; Swaminathan et al., 2016). Colons with cecum attached were removed and placed in warmed Kreb’s solution. The mesentery was cut away, and colons were placed in an organ bath, pinned down at the cecum and distal colon end with care to not impede expulsion of contents. The organ bath was kept at 37°C and filled with circulating warmed Kreb’s solution (NaCl, 120.9 mM; KCl, 5.9 mM; NaHCO_3_, 25.0 mM; Monobasic NaH_2_PO_4_, 1.2 mM; CaCl_2_, 3.3 mM; MgCl_2_•6H_2_0, 1.2 mM; D-Glucose, 11.1 mM) saturated with carbogen (95% O_2_ and 5% CO_2_). Colons were allowed to acclimate for 10 minutes in the bath. Colonic motility was recorded *ex-vivo* using a high-resolution monochromatic firewire industrial camera (The Imaging Source, DMK41AF02) mounted directly above the organ bath as previously described (Spear et al., 2018; Swaminathan et al., 2016).

#### Motility Monitor – Natural Colonic Behavior

After a 10-minute acclimation period and additional 20-minutes to allow for clearing of natural fecal pellets, motility of the empty colon was recorded for a 10-minute period. Recorded videos were converted to spatiotemporal maps (STM) using Scribble 2.0 and Matlab (2012a) plugin Analyse 2.0 and annotated to determine characteristics of CMMCs (Swaminathan et al., 2016).

#### Motility Monitor – Artificial Pellet Assay

Dissection was performed as described in “*Ex-vivo* Colonic Motility Assays”, with cecum removed. Artificial pellet assay was adapted from (Costa et al., 2021). Very gently, the colon was flushed of endogenous fecal matter using warmed Kreb’s solution. After 10 min of acclimation, a 2 mm 3D-printed pellet lubricated with KY jelly was inserted through the proximal colon and gently pushed to the proximal-mid colon junction using a blunt-ended gavage needle. Colonic activity was recorded until the pellet was fully expelled from the distal end of the colon. After at least 3 successful trials in which the artificial pellet traveled through the colon independently and was fully expelled, 10 additional minutes of empty colonic activity were recorded to ensure normal behavior. Time to expulsion was determined and a trace of the pellet’s path was made using the FIJI plug-in TrackMate (v7.6.1), from which pellet velocity and max speed were determined (Ershov et al., 2021; Tinevez et al., 2017). STMs were generated as described above.

### Statistical Analysis

Statistical analyses were performed using GraphPad Prism (Version 9.4.1) with a 95% confidence limit (p < 0.05). Data are presented as mean ± SEM and checked for normal distribution. Unless otherwise noted, an unpaired t-test was used for comparison between two groups. For comparison between two or more groups, one-way or two-way analysis of variance (ANOVA) was used with Tukey’s multiple comparisons test. Experimenter and analyzer were blinded to the genotype when feasible and appropriate. “n” refers to the number of animals tested, unless otherwise stated.

## Results:

**Figure 1.**
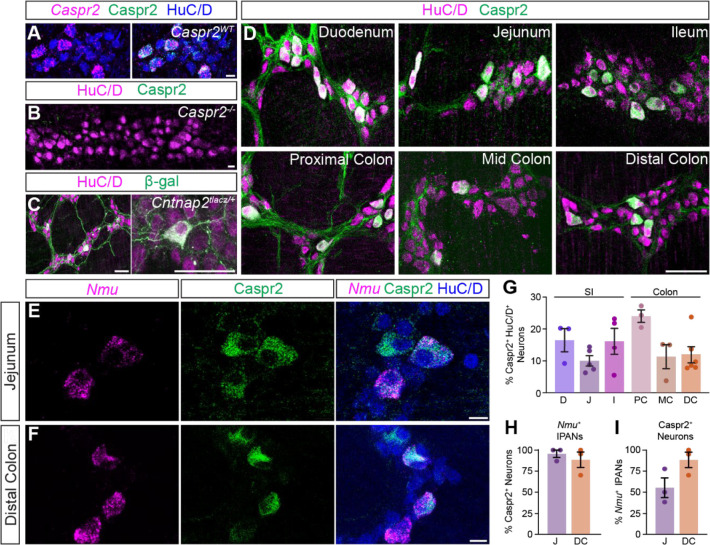
Caspr2 is expressed in sensory neurons in the small intestine and colon. **(A)** Caspr2 (green) colocalizes with *Caspr2* (magenta) transcript in adult jejunum myenteric plexus. Enteric neurons labeled with HuC/D (blue). **(B)** Caspr2 (green) is absent from HuC/D^+^ (magenta) enteric neurons in *Caspr2*^*−/−*^ myenteric plexus of adult jejunum. **(C)** β-gal (green) expression in HuC/D^+^ (magenta) neurons of adult *Cntnap2*^*tlacZ/+*^ jejunum. **(D)** Caspr2 (green) is expressed in a subset of HuC/D^+^ (magenta) neurons throughout the small intestine and colon. **(E, F)** A subset of *Nmu*^*+*^ (magenta) sensory neurons expresses Caspr2 (green) in the jejunum (E) and distal colon (F). **(G)** Quantification of Caspr2^+^ HuC/D^+^ neurons in the small intestine (D: 16 ± 4% [n = 3]; J: 10 ± 2% [n = 5]; I: 16 ± 4% [n = 4]) and colon (PC: 24 ± 2% [n = 3]; MC: 11 ± 4% [n = 3]; DC: 12 ± 3% [n = 6]). **(H)** The majority of *Nmu*^+^ IPANs express Caspr2 in the jejunum (96 ± 4% [n = 3]) and distal colon (89 ± 9% [n = 3]). **(I)** The majority of Caspr2^+^ neurons express *Nmu* in the jejunum (55 ± 12 % [n = 3]) and distal colon (88 ± 9% [n = 3]). Scale bars, (A, B, E, F) 10 μm, (C, D) 50 μm. D: Duodenum, J: Jejunum, I: Ileum, PC: Proximal colon, MC: Mid colon, DC: Distal colon.

To define the distribution of Caspr2 in the mouse intestines, we used an antibody against Caspr2, which we validated using *Caspr2* transcript co-expression (Figure 1A) and *Caspr2*^*−/−*^ mice (Figure 1B) (Poliak et al., 2003). As we were interested in querying the role of Caspr2 in GI motility, we focused our analysis on the myenteric plexus, which harbors most of the circuitry required for motility (Spencer & Hu, 2020). We further examined *Caspr2*^*tlacz/+*^ mice (Gordon et al., 2016), and found β-gal-expressing neurons and projections throughout the SI and colon (Figure 1C and data not shown).

The expression of neurotransmitters and neuromodulators can differ between intestinal regions (Hamnett et al., 2022). Caspr2 is expressed in a subset of HuC/D^+^ enteric neurons throughout the entire length of the adult mouse intestines. We assessed Caspr2 expression in distinct regions of the SI (duodenum, jejunum, and ileum) and colon (proximal, mid, and distal) (Figure 1D). We found Caspr2 expression in 10–25% of HuC/D^+^ enteric neurons, depending on the region analyzed (Figure 1G). We further observed Caspr2 expression in 5-HT^+^ intestinal epithelial cells, suggesting that Caspr2 is also present in a subset of enterochromaffin cells along the epithelial layer (Figure S1A) and in a small subset of Sox10^+^ progenitor/glial cells (Morarach et al., 2021) (Figure S1B).

We next asked whether Caspr2 expression in the myenteric ENS was confined to a particular neuronal subtype. We focused this part of the analysis on the distal colon due to its association with the propulsion of formed fecal pellets, and for comparison, chose the jejunum as a region within the SI. scRNA-sequencing studies of the mouse ENS have reported low expression of Caspr2 in all myenteric enteric neuronal subtypes, but high Caspr2 expression in putative sensory neuron populations in both the SI and colon (Drokhlyansky et al., 2020; Morarach et al., 2021; Zeisel et al., 2018). Intrinsic enteric sensory neurons, known as IPANs, make up approximately 26% of enteric neurons in the SI and have Dogiel Type II morphology, based on their large and smooth cell bodies and two or more long axons (Qu et al., 2008). We observed that the majority of Caspr2^+^ neurons were large in shape with smooth cell bodies (Figure S1A, C, D). We further assessed Caspr2 expression in IPANs, using *Nmu* transcript as a marker for sensory neurons (Morarach et al., 2021) (Figure 1E, F). Over 80% of *Nmu*^*+*^ neurons in both SI and colon co-expressed Caspr2 (Figure 1H) and approximately half of Caspr2^+^ neurons in the SI and 80% Caspr2^+^ neurons in the colon colocalized with *Nmu* (Figure 1I). These results suggest that Caspr2 has a subtype and region-specific expression profile, with almost all Caspr2^+^ neurons in the colon being IPANs.

**Figure 2.**
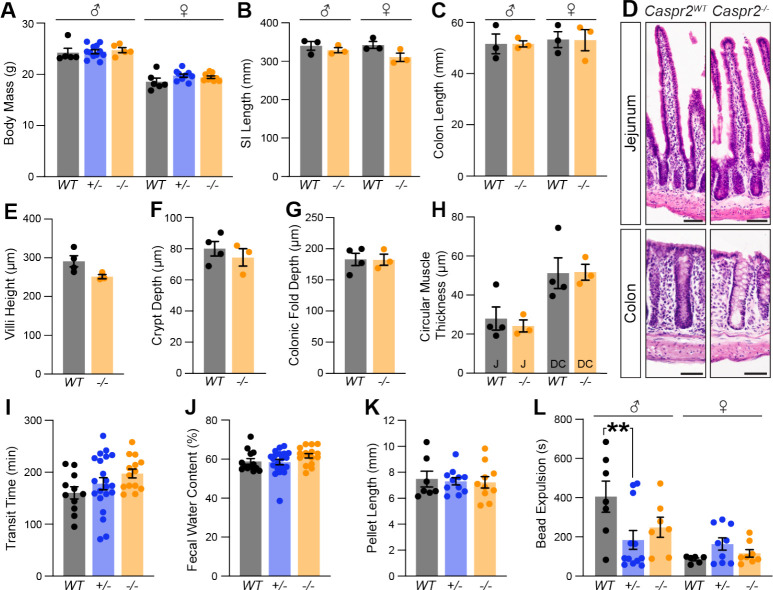
Male *Caspr2* mutant mice exhibit faster colonic bead expulsion, while gross morphology and whole GI transit remain unchanged. **(A)** Body mass is the same in *Caspr2*^*WT*^, *Caspr2*^+/−^ and *Caspr2*^−/−^ male (WT: 24 ± 0.9 g [n = 5]; +/−: 24 ± 0.4 g [n = 12]; −/−: 25 ± 0.5 g [n = 5]) and female (WT: 18 ± 0.7 g [n = 6]; +/−: 20 ± 0.3 g [n = 9]; −/−: 19 ± 0.2 g [n = 9]) mice. Two-way ANOVA: sex, F_(1,40)_= 182, p < 0.001; genotype, F_(2,40)_= 1.21, *P =* 0.31; interaction, F_(2,40)_= 0.58, *P =* 0.56. **(B)** Length of small intestine is the same in *Caspr2*^*WT*^ and *Caspr2*^−/−^ male (WT: 340 ± 11 mm [n = 3]; −/−: 329 ± 7 mm [n = 3]) and female (WT: 343 ± 9 mm [n = 3]; −/−: 311 ± 11 mm [n = 3]) mice. Two-way ANOVA: sex, F_(1,8)_= 0.69, *P =* 0.43; genotype, F_(1,8)_= 5.07, *P =* 0.05; interaction, F_(1,8)_= 1.15, *P =* 0.31. **(C)** Length of colon is the same in *Caspr2*^*WT*^ and *Caspr2*^−/−^ male (WT: 52 ± 4 mm [n = 3]; −/−: 52 ± 1 mm [n = 3]) and female (WT: 53 ± 3 mm [n = 3]; −/−: 53 ± 4 mm [n = 3]) mice. Two-way ANOVA: sex, F_(1,8)_=0.23, *P =* 0.64; genotype, F_(1,8)_= 0.001, *P =* 0.98; interaction, F_(1,8)_=0.001, *P =* 0.98. **(D)** H&E stained cross sections of jejunum and colon from *Caspr2*^*W*T^ and *Caspr2*^*−/−*^ mice. **(E)** Villi height is the same in *Caspr2*^*WT*^ (291 ± 14 μm [n = 4]) and *Caspr2*^−/−^ (252 ± 6 μm [n = 3]) jejunum. Unpaired t-test, *P =* 0.07. **(F)** Crypt depth is the same in *Caspr2*^*WT*^ (80 ± 5 μm [n = 4]) and *Caspr2*^−/−^ (74 ± 6 μm [n = 3]) mice. Unpaired t-test, *P =* 0.47. **(G)** Depth of colonic folds is the same in *Caspr2*^*WT*^ (183 ± 10 μm [n = 4]) and *Caspr2*^−/−^ (183 ± 9 μm [n = 3]) mice. Unpaired t-test, *P =* 0.97. **(H)** Circular muscle thickness is the same in *Caspr2*^*WT*^ and *Caspr2*^−/−^ jejunum (WT: 28 ± 6 μm [n = 4]; −/−: 24 ± 3 μm [n = 3]) and distal colon (WT: 51 ± 8 μm [n = 4]; −/−: 52 ± 4 μm [n = 3]). Unpaired t-tests, p_J_ = 0.63, p_DC_ = 0.96. **(I)** Whole GI transit time is the same in *Caspr2*^*WT*^ (161 ± 12 min [n = 11]), *Caspr2*^+/−^ (178 ± 12 min [n = 21]) and *Caspr2*^−/−^ (198 ± 8 min, [n = 14]) mice. One-way ANOVA, F_(2,43)_= 0.129, *P =* 0.13. **(J)** Fecal water content is the same in 9 week old *Caspr2*^*WT*^ (59 ± 2% [n = 13])*, Caspr2*^+/−^ (59 ± 1% [n = 20]) and *Caspr2*^−/−^ (62 ± 1% [n = 16]) mice. One-way ANOVA, F_(2,46)_ = 1.766, *P =* 0.18. **(K)** Fecal pellet length is the same iin 9 week old *Caspr2*^*WT*^ (7.5 ± 0.6 mm [n = 7]), *Caspr2*^+/−^ (7.3 ± 0.3 mm [n = 11]) and *Caspr2*^−/−^ (7.2± 0.4 mm [n = 10]) mice. One-way ANOVA, F_(2,25)_=0.08, *P =* 0.08. **(L)** Time to bead expulsion is shorter in male *Caspr2*^+/−^ mice compared to *Caspr2*^*WT*^ mice (WT: 405 ± 79.5 s [n = 7]; +/−: 185 ± 48 s [n = 12]; −/−: 249 ± 51 s [n = 7]). Bead expulsion time in female *Caspr2*^*WT*^ *, Caspr2*^*+/−*^ and *Caspr2*^−/−^ mice is unchanged (WT: 86 ± 7 s [n = 6]; 164 ± 32 s [n = 9]; −/−: 116 ± 20 s [n = 8]). Two-way ANOVA: sex, F_(1,43)_ = 16.06, p < 0.001; genotype, F_(2,43)_=1.22, *P =* 0.31; interaction, F_(2,43)_=4.90, *P =* 0.01. All mice were 11 weeks old unless stated otherwise. Tukey’s multiple comparison test: * p < 0.05, ** p < 0.01, *** p < 0.001. Scale bar, 50 μm. WT: *Caspr2*^*WT*^; +/− : *Caspr2*^*+/−*^; −/− : *Caspr2*^*−/−*^; SI: Small Intestine; J: Jejunum; DC: Distal Colon.

We next assessed whether *Caspr2* deletion impacts body weight and GI tract morphology in a *Caspr2*^*−/−*^ mouse line (Poliak et al., 2003) and found them to be unchanged in *Caspr2*^*−/−*^ mice compared to *Caspr2*^*WT*^ littermate controls (Figure 2A–C). Given the albeit sparse epithelial expression of Caspr2, we next asked whether deletion of *Caspr2* affects the morphology of the intestinal wall (Figure 2D). We found no changes in villi height, crypt depth, and circular muscle thickness in the SI, and depth of colonic folds and colonic circular muscle thickness in *Caspr2*^*WT*^ and *Caspr2*^*−/−*^ mice (Figure 2E–H).

IPANs are considered to be critical for initiating GI motility (Furness et al., 2004). Therefore, we asked whether the absence of Caspr2 results in altered GI function. Since heterozygous mutations of *CASPR2* can lead to symptoms associated with ASD in human patients (Poot, 2017; Rodenas-Cuadrado et al., 2014; Vogt et al., 2018), we included *Caspr2*^*+/−*^ mice in this analysis. To assess whole GI transit time, we measured the length of time needed for a carmine red mixture gavaged into the stomach of the mouse to be expelled as a red fecal pellet (Spear et al., 2018). We observed no significant difference in GI transit time when comparing *Caspr2*^*WT*^, *Caspr2*^*+/−*^ and *Caspr2*^*−/−*^ mice (Figure 2I).

We next analyzed separate measures of SI and colon function, with a particular emphasis on the colon due to the high expression of Caspr2 in colonic IPANs. We examined gastric emptying and SI function using rhodamine B dextran, but found no changes in *Caspr2* mutants (Figure S2A, B). Fecal water content (Spear et al., 2018) and pellet length (Swaminathan et al., 2019) were also unchanged between *Caspr2*^*WT*^*, Caspr2*^*+/−*^, and *Caspr2*^*−/−*^ mice (Figure 2J, K). To assess colonic motility, we performed a bead expulsion assay during which a glass bead was inserted into the distal colon from the anal end and the amount of time to expulsion was recorded (Spear et al., 2018; Treichel et al., 2022). The average time to bead expulsion was unchanged in *Caspr2* mutants. However, when separating the mice by gender, we observed that *Caspr2*^*+/−*^ male mice expelled the bead 50% faster when compared to *Caspr2*^*WT*^ littermates (Figure 2L). The average time to expulsion in *Caspr2*^−/−^ male mice was also faster, however this finding was not statistically significant.

**Figure 3.**
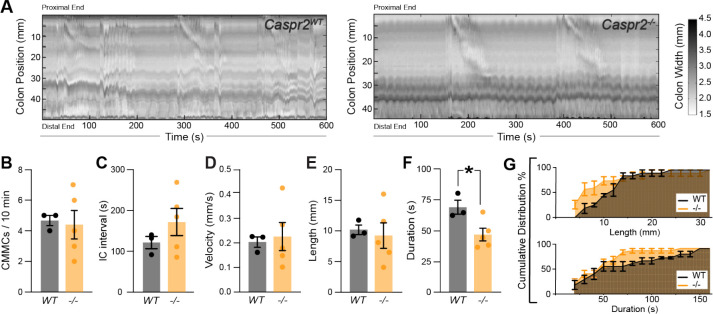
Shorter-lasting colonic migrating motor complexes (CMMCs) in the empty colon of *Caspr2* mutant mice. **(A)** Representative spatiotemporal maps of 10 minute video recordings from *Caspr2*^*WT*^ and *Caspr2*^*−/−*^ empty colons. Color scale indicates colonic diameter. **(B)** Number of CMMCs are the same in *Caspr2*^*WT*^ (4.7 ± 0.3 [n = 3]) and *Caspr2*^−/−^ (4.4 ± 0.9 [n = 5]) mice. Unpaired t-test, *P =* 0.84. **(C)** Intra-contraction interval is unchanged in *Caspr2*^−/−^ (172 ± 33 s [n = 5]) compared to *Caspr2*^*WT*^ (122 ± 15 s [n = 3]) mice. Unpaired t-test, *P =* 0.31. **(D)** CMMC velocity is the same in *Caspr2*^*WT*^ (0.21 ± 0.02 mm/s [n = 3]) and *Caspr2*^−/−^ (0.23 ± 0.06 mm/s [n = 5]) mice. Unpaired t-test, *P =* 0.78. **(E)** Length of CMMCs is the same in *Caspr2*^*WT*^ (10.2 ± 0.7 mm, [n = 3]) and *Caspr2*^−/−^ (9.3 ± 2.1 mm [n = 5]) mice. Unpaired t-test, *P =* 0.75. **(F)** CMMCs are shorter-lasting in *Caspr2*^−/−^ (47.6 ± 5.2 s [n = 5]) compared to *Caspr2*^*WT*^ (70.2 ± 5.8 s [n = 3]) mice. Unpaired t-test, *P =* 0.03. **(G)** Cumulative distributions for CMMC duration (s) and length (mm) in *Caspr2*^*WT*^ and *Caspr2*^*−/−*^ mice. IC: Intra-contraction interval.

To further assess colonic motility in *Caspr2*^*−/−*^ mice we used an *ex-vivo* motility monitor. In this setup, the colon is isolated from extrinsic CNS innervation, allowing us to focus on the effect of loss of ENS-intrinsic Caspr2 (Swaminathan et al., 2016). We generated spatiotemporal maps (STMs) of the recorded *ex-vivo* motility (Figure 3A) and observed that CMMCs were 31% shorter-lasting in *Caspr2*^*−/−*^ mice compared to *Caspr2*^*WT*^ mice (Figure 3F, G). CMMC number, intra-contraction interval, velocity and length remained unchanged (Figure 3B–E).

**Figure 4.**
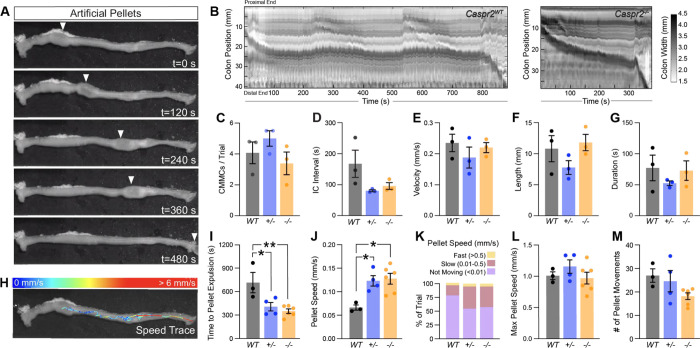
Artificial pellets expel faster from *Caspr2* mutant colon in *ex vivo* motility monitor. **(A)** Time series images of artificial pellet traversing the colon over time. White arrowheads indicate center of artificial pellet. **(B)** Representative spatiotemporal maps of full-length artificial pellet trials from *Caspr2*^*WT*^ and *Caspr2*^*−/−*^ mice. Color scale indicates colonic diameter. **(C)** Number of CMMCs during trial period are similar between *Caspr2*^*WT*^ (4.1 ± 0.7 [n = 3]), *Caspr2*^*+/−*^ (5 ± 0.5 [n = 3] and *Caspr2*^−/−^ (3.4 ± 0.7 min [n = 3]) mice. One-way ANOVA: F_(2,6)_ = 1.52, *P =* 0.29. **(D)** Intra-contraction interval is not statistically different between *Caspr2*^*WT*^ (167 ± 44 s [n = 3]), *Caspr2*^*+/−*^ (81 ± 3 s [n = 3]) and *Caspr2*^−/−^ (96 ± 11 s [n = 3]) mice. One-way ANOVA: F_(2,6)_ = 3.12, *P =* 0.12. **(E)** Velocity of CMMCs is the same in *Caspr2*^*WT*^ (0.23 ± 0.03 mm/s [n = 3]), *Caspr2*^*+/−*^ (0.19 ± 0.03 mm/s [n = 3]) and *Caspr2*^−/−^ (0.22 ± 0.02 mm/s [n = 3]) mice. One-way ANOVA: F_(2,6)_ = 0.78, *P =* 0.5. **(F)** Length of CMMCs is the same in *Caspr2*^*WT*^ (10.8 ± 2.1 mm [n = 3]), *Caspr2*^*+/−*^ (7.8 ± 1.1 mm [n = 3]) and *Caspr2*^−/−^ (11.8 ± 1.3 mm [n = 3]) mice. One-way ANOVA: F_(2,6)_ = 1.79, *P =* 0.25. **(G)** Duration of CMMCs is the same in *Caspr2*^*WT*^ (77 ± 20 s [n = 3]), *Caspr2*^*+/−*^ (52 ± 4 s [n = 3]) and *Caspr2*^−/−^ (73 ± 16 s [n = 3]) mice. One-way ANOVA: F_(2,6)_ = 0.76, *P =* 0.51. **(H)** Trace of pellet path with speed of pellet represented by color scale. **(I)** Time to pellet expulsion is shorter in *Caspr2*^*+/−*^ (407 ± 54 s [n = 4]) and *Caspr2*^*−/−*^ mice (350 ± 28 s, [n = 6]) compared to *Caspr2*^*WT*^ (714 ± 130 s [n = 3]) mice. One-way ANOVA: F_(2,10)_ = 8.72, *P =* 0.006. **(J)** Speed of artificial pellet is the same in *Caspr2*^*WT*^ (0.07 ± 0.01 mm/s, [n = 3]), *Caspr2*^*+/−*^ (0.12 ± 0.01 mm/s, [n = 4]) and *Caspr2*^*−/−*^ (0.13 ± 0.01 mm/s [n = 6]) mice. One-way ANOVA: F_(2,10)_=7.46, *P =* 0.01. **(K)** *Caspr2*^*+/−*^ (55 ± 3 % [n = 4]) and *Caspr2*^*−/−*^ (58 ± 5 % [n = 6]) mice spend less time not moving compared to *Caspr2*^*WT*^ (79 ± 3 % [n=3]) mice. < 0.01 mm/s (WT: ; +/− : 55 ± 3 % [n = 4]; −/−: 58 ± 5 % [n = 6]). One-way ANOVA for, F_(2,10)_=6.41, *P =* 0.02. **(L)** Max speed of pellet is the same in *Caspr2*^*WT*^ (1.01 ± 0.06 mm/s, [n = 3]), *Caspr2*^*+/−*^ (1.16 ± 0.11 mm/s, [n = 4]) and *Caspr2*^*−/−*^ (0.97 ± 0.09 mm/s, [n = 6]) mice. One-way ANOVA: F_(2,10)_ = 1, *P =* 0.4. **(M)** Number of pellet movement intervals is unchanged in *Caspr2*^*WT*^ (27 ± 3 [n = 3]), *Caspr2*^*+/−*^ (25 ± 5 [n = 4]) and *Caspr2*^*−/−*^ (18 ± 1 [n = 6]) mice. One-way ANOVA: F_(2,10)_ = 2.62, *P =* 0.12. Tukey’s multiple comparison test: * p < 0.05, ** p < 0.01, *** p < 0.001. IC: Intra-contraction interval; WT: *Caspr2*^*WT*^; +/− : *Caspr2*^*+/−*^; −/− : *Caspr2*^*−/−*^

Given that IPANs are thought to respond to stretch, we next assessed *ex-vivo* colonic motility in response to a stimulus. We inserted a natural-shaped 3D-printed artificial fecal pellet through the proximal colon and recorded colonic behavior until complete expulsion of the pellet from the distal colon (Costa et al., 2021). The artificial pellet served as a normalized stimulus that was able to travel the entire length of the mid and distal colon (Figure 4A). STMs revealed that the number, velocity, length, and duration of CMMCs were the same in *Caspr2*^*WT*^, C*aspr2*^*+/−*^ and *Caspr*^*-−/−*^ mice (Figure 4B,C,E,F). The intra-contraction interval appeared shortened in C*aspr2*^*−/−*^ mice, however this finding was not statistically significant (Figure 4D).

We next analyzed the propulsion of the artificial pellet through the colon. The time needed to expel the pellet was shortened by 43% in *Caspr2*^*+/−*^ and by 51% in *Caspr2*^*−/−*^ mice compared to *Caspr2*^*WT*^ littermate controls (Figure 4I). Using TrackMate (v7.6.1) (Ershov et al., 2021; Tinevez et al., 2017) to create a trace of the pellet’s movement (Figure 4H), we found that the pellet moved twice as fast in *Caspr2*^*+/−*^ and *Caspr2*^*−/−*^ compared to *Caspr2*^*WT*^ mice (Figure 4J). When analyzing pellet speed per trial, we found that the pellet spends less time not moving (Figure 4K). There was no significant difference in maximum pellet speed or in the number of pellet movements (Figure 4L, M). These findings suggest that in the presence of luminal content, colonic transit is accelerated in *Caspr2* mutants.

**Figure 5.**
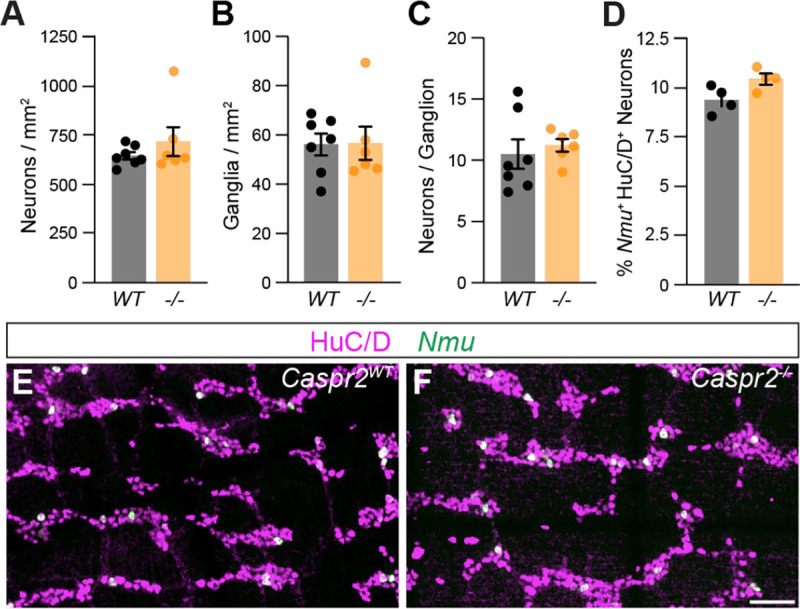
ENS organization is normal in *Caspr2* mutant distal colon. **(A)** Number of HuC/D^+^ neurons is the same in *Caspr2*^*WT*^ (648 ± 19 [n = 7]) and *Caspr2*^*−/−*^ (721 ± 74 [n = 6]) mice. Unpaired t-test, *P =* 0.32. **(B)** Number of enteric ganglia is the same in *Caspr2*^*−/−*^ (57 ± 7 [n = 6]) compared to *Caspr2*^*WT*^ (56 ± 4 [n = 7]) mice. Unpaired t-test, *P =* 0.95. **(C)** Number of neurons per ganglion is unchanged in *Caspr2*^*−/−*^ (11 ± 1 [n = 6]) compared to *Caspr2*^*WT*^ (11 ± 1 [n = 7]) mice. Unpaired t-test, *P =* 0.62. **(D)** Percent of *Nmu*^+^ HuC/D^+^ neurons is unchanged in *Caspr2*^*−/−*^ (10.5 ± 0.3 [n = 4]) compared to *Caspr2*^*WT*^ (9.4 ± 0.3 [n = 4]) mice. Unpaired T-test, *P =* 0.05. **(E, F)** *Nmu* (green) is expressed in a subset of HuC/D^+^ (magenta) neurons in *Caspr2*^*WT*^ (E) and *Caspr2*^*−/−*^ (F) distal colon. Scale bar, 50 μm.

Since changes in GI motility have been reported to result from altered ENS organization (Margolis et al., 2016), we next assessed the neuronal organization in the distal colon. The total number of HuC/D^+^ myenteric neurons in the distal colon was unchanged in *Caspr2*^*−/−*^ mice (Figure 5A). We additionally analyzed the distribution of enteric neurons into ganglia by quantifying the number of enteric ganglia, the number of neurons within a ganglion, and the number of intra-ganglionic and extra-ganglionic neurons using COUNTEN (Kobayashi et al., 2021) (Figure 5B–D, S3A,B). However, none of these parameters were changed in *Caspr2*^*−/−*^ mice. Given the predominant expression of Caspr2 in IPANs, we next asked whether lack of Caspr2 impacts IPANs, however, we found no difference in the number of *Nmu*^*+*^ neurons in *Caspr2*^*−/−*^ and *Caspr2*^*WT*^ mice (Figure 5D–F). Therefore, the overall organization of the ENS does not appear to be altered in *Caspr2*^*−/−*^ mice.

## Discussion:

GI dysfunction is a prevalent symptom in patients with ASD (Holingue et al., 2018). In this study, we aimed to determine whether the ASD-related gene, *Caspr2,* plays a role in mouse GI function by characterizing Caspr2’s expression in the intestines and assessing colonic function and ENS organization in *Caspr2*^*−/−*^ mice. Our findings reveal that Caspr2 is predominantly expressed in enteric sensory neurons and that *Caspr2*^*−/−*^ mice have altered CMMCs and accelerated colonic motility when compared to *Caspr2*^*WT*^ mice in an *ex-vivo* motility monitor. The overall organization of the ENS appears unchanged.

Sensory over-responsivity has been correlated with the presence of GI issues in children diagnosed with ASD (Mazurek et al., 2013) and Caspr2 has been linked to sensory processing deficits and hypersensitivity in both human and mouse CNS and PNS (Dawes et al., 2018; Fernández et al., 2021; Peñagarikano et al., 2011). Our finding that Caspr2 is expressed in the majority of *Nmu*^*+*^ IPANs is consistent with scRNA-seq studies reporting a high expression of Caspr2 in putative enteric sensory neuron classes (Drokhlyansky et al., 2020; Morarach et al., 2021; Zeisel et al., 2018). IPANs are thought to be critical for initiating propulsive CMMCs and downstream motility patterns (Feng et al., 2022; Fung et al., 2021; Nestor-Kalinoski et al., 2022; Spencer et al., 2018) and the observed alterations in CMMCs in *Caspr2*^*−/−*^ mice suggests a role for Caspr2 in enteric sensory function. In agreement with previous studies (Drokhlyansky et al., 2020; Morarach et al., 2021; Zeisel et al., 2018), we note that Caspr2 is also expressed in other ENS subsets, which could contribute to GI dysfunction in *Caspr2* mutants. However, given that Caspr2 is predominantly expressed in sensory neurons of the distal colon, we are interpreting the CMMC phenotype as reflecting alterations in sensory neuron function.

Recent studies have provided growing evidence for the role of ASD-related genes in GI function. Other ASD mouse models that have been used to investigate ENS organization include *Slc6a4*^*−/−*^ (SERTKO) mice, SERT Ala56 mice (common SERT variant), *Nlgn3*^*−/−*^ mice, and *NL3*^*R451C*^ mice (human neuroligin-3 R451C missense mutation) (Hosie et al., 2019; Leembruggen et al., 2020; Margolis et al., 2016). Three out of these four mouse models show changes to the ENS and all mutants demonstrate altered GI function. SERT Ala56 mice have a hypoplastic ENS and SERTKO mice have a hyperplastic ENS (Margolis et al., 2016), both resulting in slower colonic motility. *NL3*^*R451C*^ mice have an increased number of neurons in the SI (Hosie et al., 2019; Leembruggen et al., 2020), while *Nlgn3*^*−/−*^ mice have overall normal numbers of enteric neurons. We report accelerated colonic motility in *Caspr2*^*−/−*^ mice, while the total number of neurons and organization of the ENS remains unchanged. Further investigation is needed to assess whether the connectivity and function, particularly of colonic IPANs, is altered in *Caspr2*^*−/−*^ mice.

While Caspr2 was first identified as playing a role in the longitudinal movement of potassium channels in the juxtaparanodal regions of myelinated axons (Poliak et al., 1999), considering Caspr2’s described roles and functions in other systems may shed light on its mechanisms of action in the unmyelinated ENS. Caspr2 regulates the excitability of DRG sensory neurons by altering Kv1 channel function (Dawes et al., 2018). The absence of Caspr2 leads to a reduction in overall expression of Kv1.2 channels at the soma membrane of DRG neurons, resulting in altered electrical properties and increased neuronal excitability (Dawes et al., 2018). The gene encoding Kv1.2 (*Kcna2*) is also highly expressed in colonic sensory neurons (Drokhlyansky et al., 2020). Similar to the altered cerebellar response to somatosensory stimuli previously reported in *Caspr2*^*−/−*^ mice (Fernández et al., 2021), IPANs may become hyperexcitable when activated in the absence of Caspr2, resulting in altered CMMCs and a shorter time to expulsion during the artificial pellet assay. Future experiments using isometric force transducers to measure contraction strength and calcium imaging will be needed to determine if changes in contraction strength might contribute to the faster pellet expulsion in the *Caspr2*^*−/−*^ mice.

By demonstrating altered GI motility, the *Caspr2*^*−/−*^ mouse model contributes to our understanding of the relationship between ASD and GI dysfunction. Our findings show that, in addition to its previously-described roles in the CNS and PNS, Caspr2 is expressed in enteric sensory neurons and *Caspr2*^*−/−*^ mice display changes in colonic motility. We postulate that similar mechanisms underlying sensory dysfunction in individuals with ASD may be involved in disrupting GI motility. Our findings therefore may have important implications for the diagnosis and treatment of GI symptoms in patients with ASD, particularly in those that present with sensory processing abnormalities.

## Supplementary Material

Supplement 1
