# Supplementary material for: Loss of ASD-Related Molecule Cntnap2 Affects Colonic Motility in Mice
Source: bioRxiv. 2023 Sep 1:2023.04.17.537221. Preprint. [Version 3] doi: 10.1101/2023.04.17.537221 (PMC10153124; doi:10.1101/2023.04.17.537221)
Supplement: Supplement 1 [file media-1.pdf]

Supplementary Material

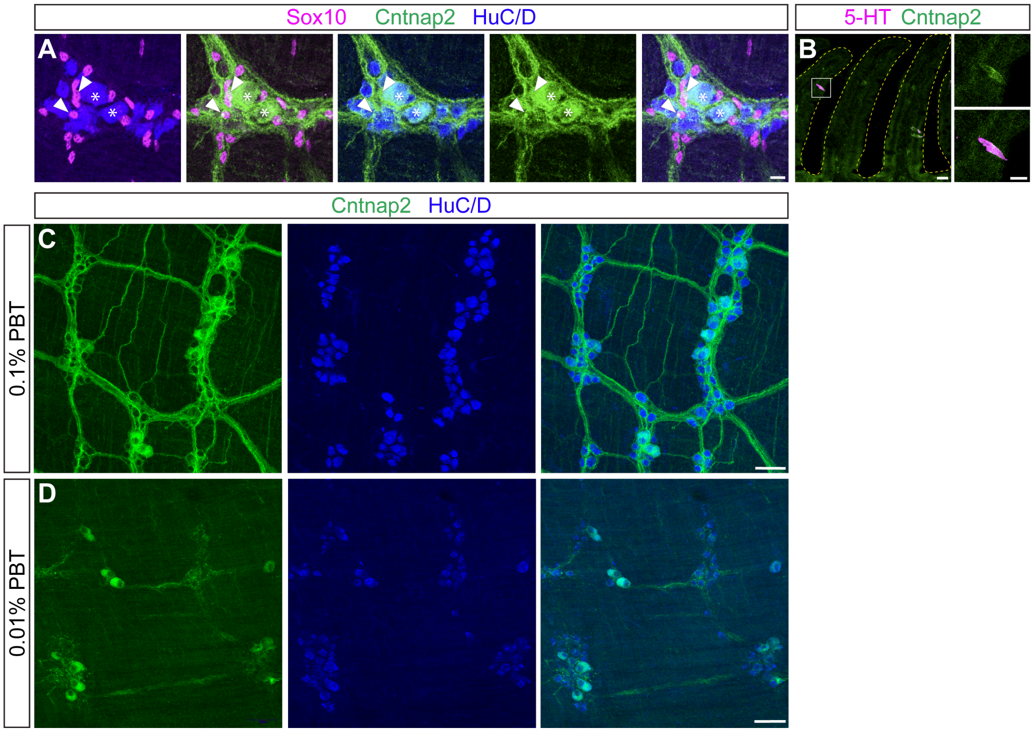

Figure S1. Cntnap2 colocalizes with a subset of progenitor/glial and enteroendocrine cells. (A) Cntnap2 (green) colocalizes with a subset of $Sox10^+$ cells (magenta) (arrowheads). Enteric neurons labeled with HuC/D (blue). $Cntnap2^+$ neurons indicated with asterisks. **(B)** Cntnap2 (green) colocalizes with a subset of $5\text{-}HT^+$ cells (magenta) in the epithelium. **(C, D)** $Cntnap2^+$ (green) cell bodies and projections are visible with 0.1% PBT (C), while cell body labeling is pronounced with 0.01% PBT (D). Scale bar, (A, B) 10 µm, (C) 50 µm.
