# Supplementary material for: Loss of ASD-Related Molecule Caspr2 Affects Colonic Motility in Mice
Source: bioRxiv. 2023 Apr 19:2023.04.17.537221. Preprint. [Version 2] doi: 10.1101/2023.04.17.537221 (PMC10153124; doi:10.1101/2023.04.17.537221)
Supplement: Supplementary file 1 [file media-1.pdf]

Supplementary Data and Legends:

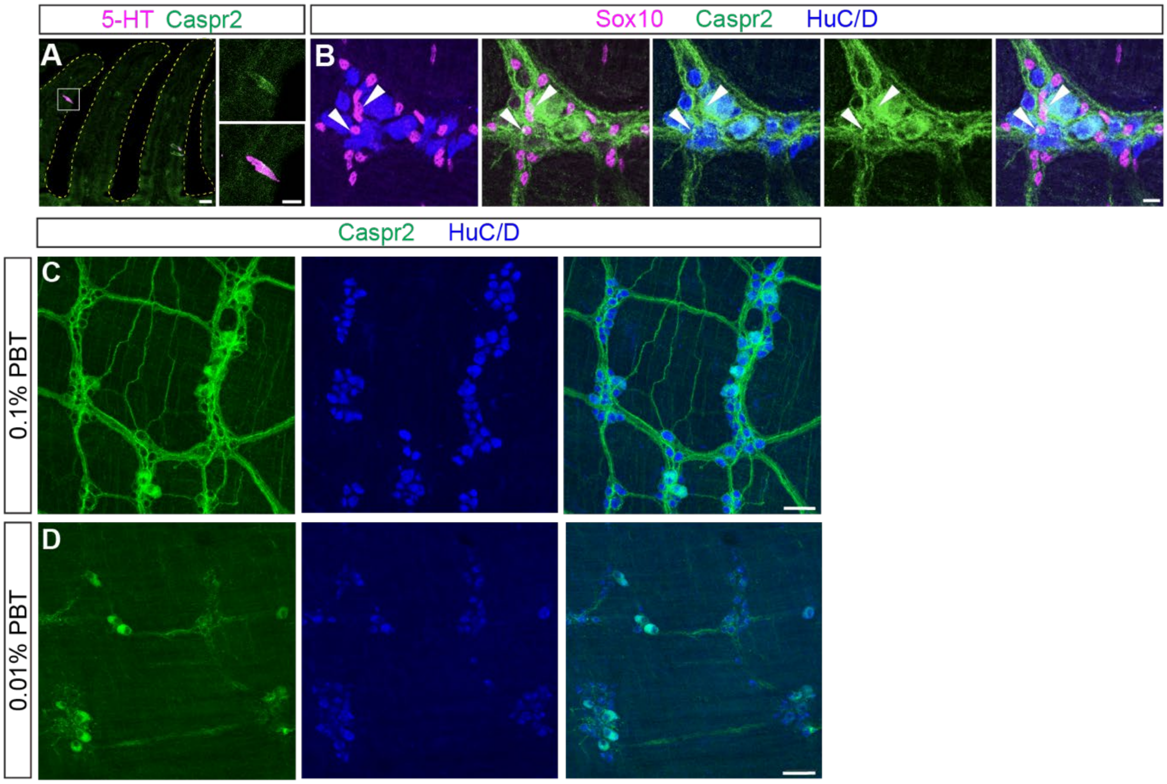

Figure S1. Caspr2 colocalizes with enteroendocrine cells and a subset of progenitor/glial.

(A) Caspr2 (green) colocalized with a subset of 5-HT⁺ cells (magenta) in the epithelium. **(B)** Caspr2 (green) (blue). **(C, D)** Caspr2⁺ (green) cell bodies and projections are visible with 0.1% PBT (C), while cell body labeling is pronounced with 0.01% PBT (D). Scale bar, (A-B) 10 µm, (C) 50 µm

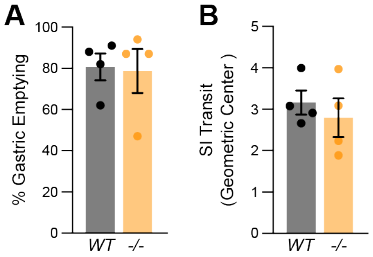

Figure S2. Gastric emptying and small intestine transit are unchanged in *Caspr2* mutant mice. **(A)** Gastric emptying is unchanged in *Caspr2*$^{-/-}$ (79 ± 11 [n = 4]) compared to *Caspr2*^{WT} (81 ± 7 [n = 4]) mice. Unpaired t-test, p = 0.88. **(B)** Small intestine transit is unchanged in *Caspr2*$^{-/-}$ (2.8 ± 0.5 [n = 4]) compared to *Caspr2*^{WT} (3.2 ± 0.3 [n = 4]) mice. Unpaired t-test, p = 0.53.

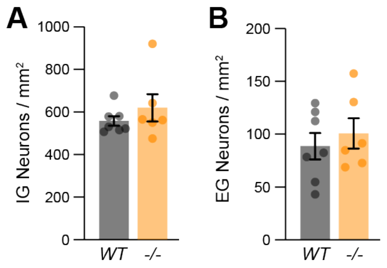

Figure S3. No difference in extra- or intra-ganglionic neuron number in *Caspr2* mutant mice. **(A, B)** Number of intra- (A) and extra-ganglionic (B) neurons in distal colon is the same in *Caspr2*^{WT} (intra: 559 ± 23, extra: 89 ± 13 [n = 7]) and *Caspr2*$^{-/-}$ (intra: 620 ± 64, extra: 101 ± 14 [n = 6]) mice. Unpaired t-test, p_{intra} = 0.36, p_{extra} = 0.54. IG: Intra-ganglionic; EG: Extra-ganglionic
